# P-113. Interim Results of the First-in-human Phase 1 Trial of AIC468: A Novel Antisense Oligonucleotide that Targets BK-Virus Transcripts

**DOI:** 10.1093/ofid/ofaf695.341

**Published:** 2026-01-11

**Authors:** Susanne Bonsmann, Ashish Soman, Vedran Pavlovic, Bernadette Surujbally, Gnana Oli Rajaraman, Cynthia Wat, Dirk Kropeit

**Affiliations:** AiCuris Anti-infective Cures AG, Wuppertal, Nordrhein-Westfalen, Germany; AiCuris Anti-infective Cures AG, Wuppertal, Nordrhein-Westfalen, Germany; AiCuris Anti-infective Cures AG, Wuppertal, Nordrhein-Westfalen, Germany; AiCuris Anti-Infective Cures AG, Bishops Stortford, England, United Kingdom; AiCuris Anti-infective Cures AG, Wuppertal, Nordrhein-Westfalen, Germany; Aicuris Anti-infective Cures AG, London, England, United Kingdom; AiCuris Anti-infective Cures AG, Wuppertal, Nordrhein-Westfalen, Germany

## Abstract

**Background:**

Reactivation of BKV in immunosuppressed patients, kidney transplant recipients, is associated with BKV-associated nephropathy, renal failure & graft loss with no effective or approved antiviral treatments. AIC468 is a splice-modulating antisense oligonucleotide (ASO) that targets BKV pre-mRNA and prevents large T-Ag formation, which is essential for viral replication. Potent antiviral activity was demonstrated in BKV-infected primary human kidney epithelial cells and the mechanism of action was confirmed in a BKV-Tat mouse model.

**Table 1:**
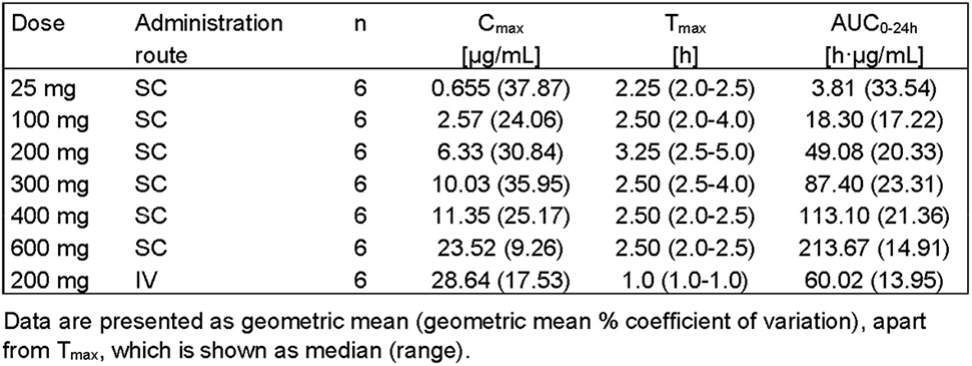
Plasma pharmacokinetic parameters after single doses of AIC468 in healthy volunteers

**Figure 1:**
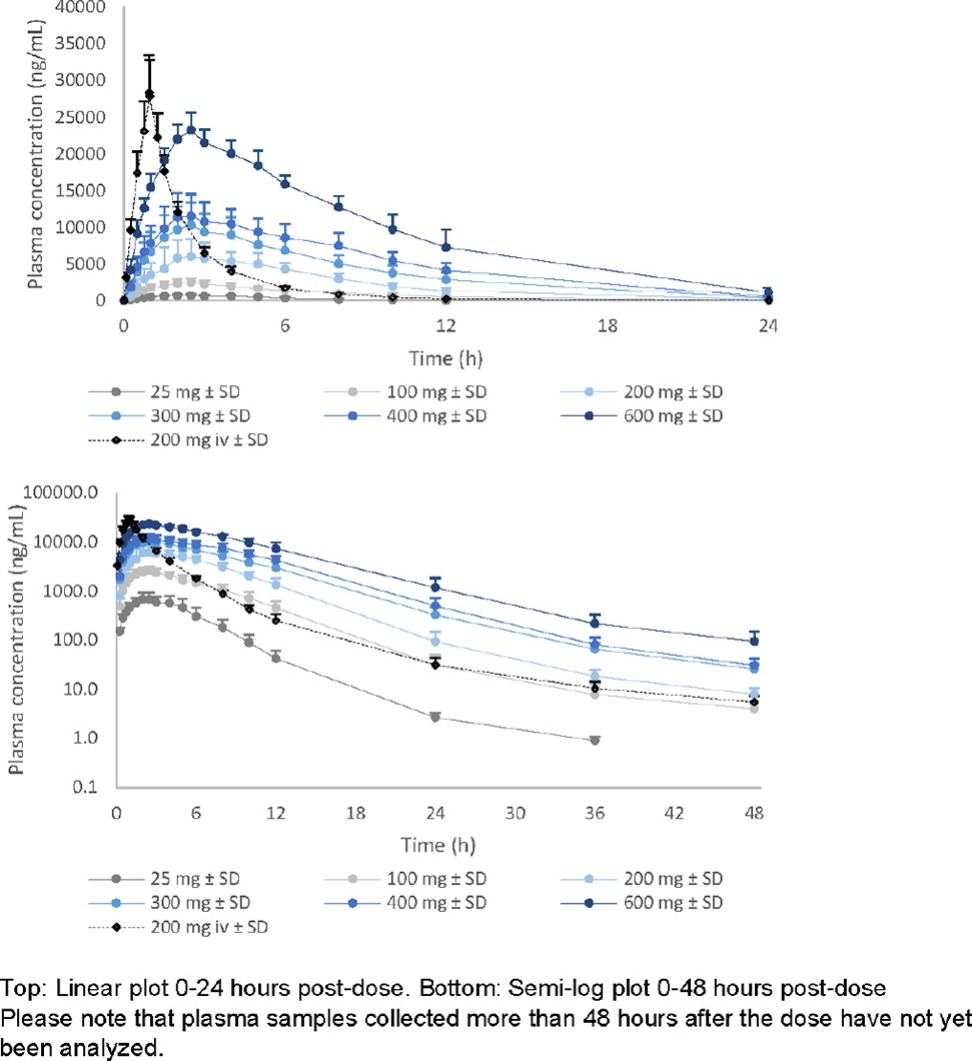
Mean (+SD) plasma concentration profiles after single doses of AIC468 in healthy volunteers (0-48 hours)

**Methods:**

This single-center, randomized, double-blind, placebo-controlled, FIH trial (EUCT 2023-510074-13-00) consists of two parts. Part A is a single ascending dose study in healthy volunteers (HVs), which includes 6 subcutaneous (SC) and 1 intravenous (IV) administration cohorts. Part B is a multiple dose ascending dose study in HVs which includes 3 SC administration cohorts with once weekly dosing over one month. Within each cohort in Part A and B, 6 HVs received AIC468 and 2 received a matching placebo. Here we report interim pharmacokinetics (PK) and blinded safety results from Part A of the ongoing trial.

**Results:**

Fifty-six HVs were dosed across 7 cohorts. A total of 93 AEs were reported in 41 (73%) HVs, of which 31 (33%) were considered related to study treatment. Majority of AEs 78 (84%) were mild in intensity, including 9 (10%) Injection Site Reactions. One unrelated SAE (hospitalization due to cut wound) was reported. There were no severe AEs, or dose-related trends in the nature, incidence or severity of AEs. Vital signs, ECG, and safety laboratory parameters were unremarkable.

Plasma exposure (AUC_0-24h_, C_max_) increased with dose from 25 to 600 mg in a mild supra-proportional manner. AIC468 showed typical ASO plasma concentration profiles with rapid absorption after SC administration (T_max_ between 2–5 hours) and rapid distribution to peripheral tissues. The absolute bioavailability at 200 mg SC is 82%.

**Conclusion:**

AIC468 single SC doses between 25 mg and 600 mg and IV dose of 200 mg were safe and well tolerated in HVs. Plasma exposure increased supra-proportionally with ascending SC doses, most likely due to saturation of tissue uptake mechanisms. Additional data from this ongoing Phase 1 trial will be presented.

**Disclosures:**

Susanne Bonsmann, Diploma in Chemistry Engineering, AiCuris Anti-infective Cures AG: Employee Vedran Pavlovic, MD, VP Pharma Consultancy Ltd: Director|VP Pharma Consultancy Ltd: Ownership Interest|VP Pharma Consultancy Ltd: Stocks/Bonds (Private Company) Bernadette Surujbally, MSc, AiCuris Anti-Infectives Cures AG: Stocks/Bonds (Private Company)|Roche: Stocks/Bonds (Private Company) Gnana Oli Rajaraman, B.Pharm, PhD, AiCuris Anti-infective Cures AG: Employee Cynthia Wat, MBBS MRCP MFPM, Aicuris Anti-infective Cures AG: Employee|Aicuris Anti-infective Cures AG: Stocks/Bonds (Private Company)|ID Pharma Consultancy Ltd: Director|ID Pharma Consultancy Ltd: Ownership Interest|ID Pharma Consultancy Ltd: Stocks/Bonds (Public Company) Dirk Kropeit, n/a, AiCuris Anti-infective Cures AG: employee

